# Photocatalytic degradation of Aniline from aqueous solutions under sunlight illumination using immobilized Cr:ZnO nanoparticles

**DOI:** 10.1038/s41598-017-01461-5

**Published:** 2017-05-03

**Authors:** Meghdad Pirsaheb, Behzad Shahmoradi, Masumeh Beikmohammadi, Esmaeel Azizi, Hooshyar Hossini, Ghulam Md Ashraf

**Affiliations:** 1 Research Center for Environmental Determinants of Health, Kermanshah University of Medical Sciences, Kermanshah, Iran; 2 Department of Environmental Health Engineering, Faculty of Health, Kurdistan University of Medical Sciences, Sanandaj, Iran; 3 Environmental Health Research Center, Kurdistan University of Medical Sciences, Sanandaj, Iran; 4 Student Research Committee, Kermanshah University of Medical Sciences, Kermanshah, Iran; 5 Department of Environmental Health Engineering, Faculty of Health, Kermanshah University of Medical Sciences, Kermanshah, Iran; 6 King Fahd Medical Research Center, King Abdulaziz University, Jeddah, Saudi Arabia

## Abstract

The present study aimed at synthesizing chromium doped zinc oxide nanoparticles (Cr:ZnO NPs) under mild hydrothermal conditions (temperature ~100 °C, p = autogenous and time ~12 hr). Chromium oxide and *n*-butylamine were used as dopant and surface modifier, respectively. The characteristics of the synthesized nanoparticles were determined through conducting specialized experiments including powder XRD, FTIR, SEM, EDX, and UV-VIS spectroscopy. Then, the Cr:ZnO NPs were immobilized on a sandblasted glass through thermal method. The photocatalytic degradation of aniline was conducted in a continuous reactor with a volume of 1.5 liters. Before and after photocatalytic degradation, the immobilized Cr:ZnO NPs were characterized for SEM and EDX to determine the degree of stability of immobilized nanoparticles as well as the influence of the current applied on them. The photodegradation operational parameters investigated were aniline initial concentration (150, 200, and 250 mg/L), pH (5, 7, 6, and 12), and reaction time (2, 4, and 6 hours) under sunlight illumination. The characterization results indicated high purity of the Cr:ZnO NPs and no change in morphology or composition even after the immobilization and photo-oxidation process. Finally, it was found that the optimum conditions for 93% removal of aniline under sunlight illumination was about 6 hours retention time at pH 9.

## Introduction

Over half a million organic compounds have been created from the beginning of the new century, with around 10,000 new compounds being invented every year^1^. Aniline is a synthesized intermediate compound with poisonous properties and has a solubility of 35000 mg/L in water. Therefore, there is a risk of its presence in both wastewater and drinking water resources. Furthermore, because it is widely used and dumped through urban sewage as well as industrial and agricultural wastewater, aniline can be accumulated in the environment^2^. Aniline or amino benzene is an organic compound with the formula C_6_H_5_NH_2_. This organic compound consists of a benzene ring attached to an amino group. Aniline is an oily and colorless compound, which is easily oxidized in air and forms a red-brown substance. Like most volatile amines, it possesses odor of rotten fish. Aniline is poisonous and ignites readily (184.4 °C), burning with a smoky flame^3^. It is a carcinogenic chemical compound and causes tumors in animals. It also increases the risk of bladder cancer among humans. According to health rules, it has a threshold limit value of 2 ppm per 8 hours for skin exposure and 5 ppm per 8 hours for airborne permissible exposure^4^. On the other hand, this compound causes fast reactions in the blood, converts hemoglobin to methaemoglobin, and leads into cyanosis. Moreover, long time exposure to this substance can harm kidneys, liver, bones, and the nervous system^5^. It can also cause anemia, loss of appetite, and weight loss. Thus, even very low concentrations of aniline in water resources can cause damage to aquatic life and human health^6^.

US Environmental Protection Agency (USEPA) and European Commodity Clearing (ECC) have classified aniline as resistant contaminants^7^. Based on USEPA’s recommendation, the maximum concentration level (MCL) of aniline in water is 5 mg/L^6^. As a raw material and the precursor for the production of polyurethane, aniline is widely used in chemical industry, especially in the manufacturing of paint, rubber, pharmaceuticals, plastics, and pesticides, with over 150 compounds being derived from it^8^. The annual production of aniline only in US and China is 80000 and 457000 tons, respectively^9^. Because of the presence of various petrochemical industries, the production of rubber and paint, and an annual demand of 156 tons for aniline in Iran^10^, large amounts of this compound are available in the production line and the effluent of such industries. The negative health and environmental effects of aniline makes its removal one of the main priorities.

In order to reduce the emissions from aniline-producing industries, modern engineering technologies must be utilized. Various methods (e.g. physical adsorption, electrochemical oxidation, biodegradation, and catalytic wet oxidation) have been exploited to remove phenolic pollutants and aniline^11^. Despite their applicability and effectiveness, these methods are inefficient because of some disadvantages involving high costs, lack of pollutant’s complete removal, and, in some cases, creation of compounds that are even more poisonous than the primary pollutants^11, 12^. As a substitute for these methods, photocatalytic degradation can be used as an advanced oxidation process (AOP) in order to oxidize organic and aromatic pollutants and convert them to low-risk or safe substances for the environment^13^. In these processes, ultraviolet radiation to the semiconductor material excites the electrons from the valence to conduction band. Electron excitation produces hydroxyl radicals in aqueous environments, a phenomenon that causes oxidation of organic compounds into low-risk and safe ones^14–16^. Zinc oxide nanoparticles are suitable for this purpose and are being widely used for AOPs^17^. Because of its unique features such as thermal stability, low cost, non-toxicity, and proper conductivity capacity, zinc oxide is applied on large scale for solar water^18^. Zinc oxide has a band-gap of 3.2 eV and a wavelength of 387 nanometers and can be excited by UV-A portion (320–380 nanometer) of chromatic radiation^17^. Because of its wide band-gap, zinc oxide is the only substance that can react to UV, which makes up only 4% of solar waves’ radiation^19^. Over the past decade, various attempts have been made to extend the broadband optical absorption to the visible light range. One of these attempts is metal and non-metal ion doping^20^. Through affecting narrow gap quantum dots of semiconductors like zinc oxide^21^, doping excites them under visible light radiation (photo-activation)^22^. Metal ion doping creates an outer layer in the energy gap. This layer is placed at the top of energy band or the bottom of conduction band, which function as the recipient or donor of electrons. Both the recipient and donor layers of electrons cause the semiconductor to react to sunlight^23^.

Different methods have been applied to synthesize zinc oxide nanoparticles and to dope metal ions into the band-gap of materials^24^, including vapor-liquid-gas process^25^, vapor-liquid-solid method^26^, soft chemistry method^27^, sol-gel method^28^, co-precipitation method^29^, and hydrothermal synthesis^30^. The majority of methods for creating nanoparticles lead to the production of larger particles, which generally require post-treatment. On the other hand, because of the presence of van der Waals forces, these particles are agglomerated and granulated^31^. Hydrothermal is a promising substitute synthesizing method since it needs low temperature and pressure. Through this procedure, it is also easy to control the size of particles. Hydrothermal method has a number of advantages over the other ones; for example, it requires simple equipment, is environmentally friendly and less harmful, and does not need purification. In this method, the features of particles can be controlled by adjusting the temperature and time of the reaction and/or the concentration of the solution^32^. One of the problems in synthesizing nanoparticles is agglomeration, granulation, and poor dispersion of particles in the environment, a phenomenon that reduces the efficiency and effectiveness of nanoparticles as suitable photocatalysts. These problems can be overcome by applying an influential strategy. A few studies have demonstrated the use of surface modifiers and proper dopants for synthesizing zinc oxide nanoparticles with special features. For example, Shahmoradi *et al*. doped ZnO with neodymium oxide and, in another study, with manganese oxide in the presence of *n*-butylamine as surface modifier^32, 33^. Their study confirmed that, in the presence of sunlight, the synthesized nanoparticles were more efficient than available pure bulky compounds^34^. Doping ZnO with indium oxide showed that there was a positive correlation between surfactant concentration and the shape and size of synthesized particles^33^. In the photocatalytic process, the catalyst can be used in suspended or immobilized form. Although suspended reactors are generally more efficient, splitting them at the end of the process takes a lot of costs and time. Hence, on a large scale, it is not economically justified. In recent years, the use of immobilized catalysts is becoming more and more fashionable^35, 36^. In their research, Jimenez *et al*. indicated that one of the problems of such reactors is water poisonousness because of the presence of nanoparticles especially in the ones used in the suspended form^37^. They believed that using proper bed for immobilized bed reactors is an effective factor in the immobilization, activity, and adhesion of the catalyst on the bed and the catalyst homogeneity. So far, numerous beds have been used for immobilizing photocatalyst, including quartz, different types of plate and curved glass, activated carbon, various kinds of ceramic, zeolites, porous rocks or pumice, glass fibers, stainless steel, and cement^38, 39^. Therefore, the aim of the present study was to synthesize immobilized chromium doped ZnO nanoparticles (Cr:ZnO NPs) and their application in photocatalytic degradation of aniline under sunlight illumination.

## Material and Methods

### Material

All chemical and regents were prepared from analytical grade.

### Synthesis of Cr:ZnO NPs

Cr:ZnO NPs were synthesized under hydrothermal conditions (T = 100 °C, P = autogenous). To do this, a 2-mole solution of zinc oxide (ZnO) (Merck, Germany) was taken as precursor. Chromium oxide (Cr_2_O_3_) was then added in predetermined concentrations (2 and 5 mole%). Then, 10 ml of 1.0 N sodium hydroxide (NaOH) was added as solvent. The solution was stirred for 5 min under laboratory conditions to obtain a homogenous mixture. After that, 1 ml *n*-butylamine was slowly added as a surfactant. After determining pH, the mixture was poured into a Teflon liner (V_fill_ = 25 mL). The prepared Teflon liner was then placed in a General-Purpose autoclave and was fully lined. Subsequently, based on the hydrothermal method, it was put in a furnace equipped with temperature control applications for 12 hours. After the passage of suitable reaction time, the autoclave was cooled at room temperature to reach thermal equilibrium with the environment. Then, the Teflon liner was removed from the autoclave and the obtained mixture was washed several times using double distilled water (EC = 0.001 Ω). After spinning off (12000 rpm for 15 minutes), the produced nanoparticles were dried at 40–50 °C temperature.

### Thermal immobilization of Cr:ZnO NPs

For this purpose, 5% suspension of the Cr:ZnO NPs was prepared and stirred over magnetic hot plate for 30 min. Then, it was washed using ultrasonic bath (ElmaP30H, Germany) by applying 50 kHz frequency of ultrasonic waves for 30 min. The aim of this stage was separating particles from each other completely and preparing them for spreading over glass plates. Then, the 10 × 18 cm sand-blasted glass plates were washed by 50% sodium hydroxide, tap water, and distilled water, sequentially. After being dried, 2.5 g of homogeneous suspension of Cr:ZnO NPs prepared through the abovementioned procedure were spread uniformly over the plates. The plates were dried at temperature of 30–40 °C. Later, the plates were put inside the furnace and the temperature reached 450 °C with an increasing rate of 5 °C/min for 2 hr^40, 41^. In order to obtain a proper thickness of immobilized nanoparticles, this action was repeated for the second and third times. Before installing and using the immobilized Cr:ZnO NPs in the reactor, the surface of glass plates was first washed by distilled water. The aim was to remove those parts of nanoparticles with improper immobilization or low stability.

### Characterization of the immobilized Cr:ZnO NPs

The characterization of the immobilized Cr:ZnO NPs was carried out using the following instruments: Powder XRD (Cu Ka, light: λ = 1.542 Ȧ, voltage: 30 kV, current: 15 mA, scan speed ~5° min^−1^, and 2θ = 10–80°) (Bruker D8 Advance machine, Germany); FTIR (SHIMADZU, model IRPrestige-21, Japan); UV-vis spectrophotometer (Elico, Minispec SL 171, Netherlands); SEM equipped with EDS (SIGMA/VP, Germany) EDS). Moreover, the size of the particles was determined using Scherrer’s equation.

### Photocatalytic degradation of aniline using immobilized ZnO NPs

This experimental study conducted was at laboratory scale with a continuous current. The variables of the study included aniline concentration (150, 200, and 250 mg/L), sunlight illumination, pH (5, 7, 9, and 12), and retention time (2, 4, and 6 hours). A continuous cube reactor was made with a capacity of 1.5 L. In order to prevent short circuit, the input was designed at the bottom and the output at the top of the reactor. In order to establish continuous current, peristaltic pumps were used (BT-100 1 L Longer pump, China). Glass plates that contained immobilized nanoparticles were placed on two sides of the cube. With the aim of increasing the efficiency of aniline oxidation process using Cr:ZnO NPs, aeration was applied to increase dissolved oxygen in wastewater, which would lead to the creation of a larger number of hydroxyl free radicals^42^. The air pump used was resumed with an air capacity of 1.5 L/min. Then, the experiments related to aniline oxidation by sunlight as the source of UV was conducted. The UV intensity of sunlight was measured using a UV meter (Hanger-Model EC1-UV-A, Switzerland) and the sunlight intensity was recorded using a Lux meter (model, company) The solvent used for extracting aniline was *n*-hexane^43^.

The aniline reading was carried out using GC-MS (Agilent-6890N) with the mass spectrometry detector (MSD) (5975 C). The flow rate of helium, as carrier gas, was 1 ml/min. The used column in this type of machine was HP-5 (internal diameter of 0.32 mm, length of 30 m, and film thickness of 0.25 micrometers). In each reading, 1 µL of sample was injected to the machine. In the injection phase, the injector’s (Intel) temperature was fixed at 250 °C and the mode of Split was 1.50. At first, the column temperature was kept at 85 °C for 3 min. Then, it was gradually increased to 240 °C with an increasing rate of 65 °C/min and was kept at this isothermal temperature for 5 min. In the axillary phase, the temperature was fixed at 280 °C. The required time for aniline exit was around 4 min. In order to let aniline and other organic compounds exit as a result of aniline oxidation, 10 min was devoted to injection^44^. The aforementioned tests were conducted according to the guidelines of the “Standard Methods for Examination of Water and Wastewater”^45^.

## Results and Discussion

**Figure 1:**
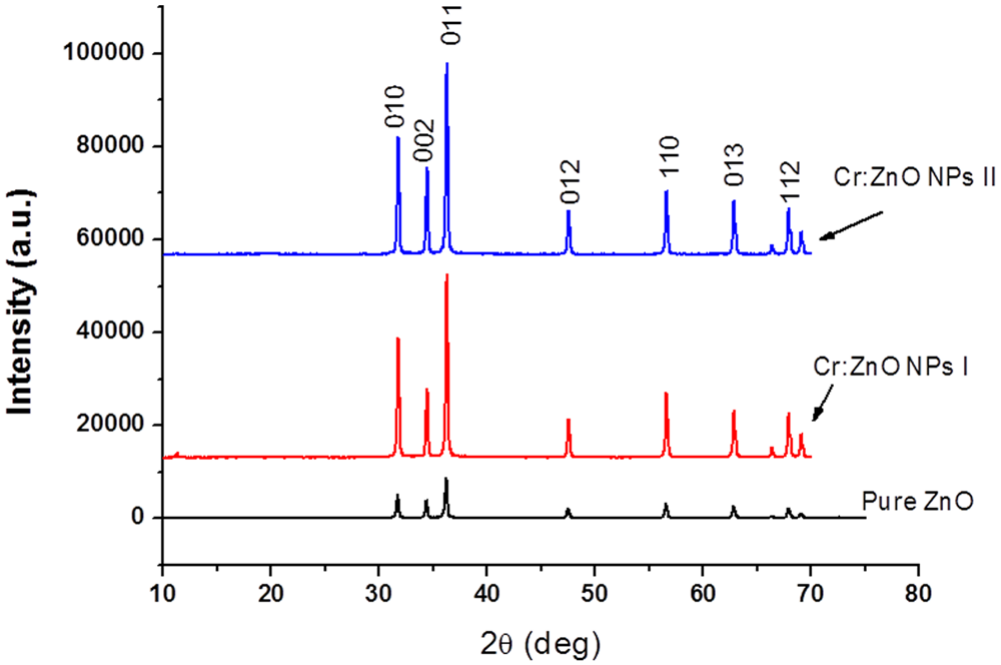
XRD powder of reagent grade zinc oxide powder, 2 mole% Cr:ZnO NPs (І), and 5 mole% Cr:ZnO NPs (П).

Figure 1 plots the Powder XRD spectra obtained from the surface of Cr:ZnO NPs. It complies with the standard sample, which can be attributed to the different diffraction pattern of ZnO^46^. The main peaks observed are at 2Ɵ of 31.73, 34.46, 36.23, 47.48, 55.65, 62.92, 67.89 degrees. On the other hand, the figure shows a crystallite hexagonal structure in which no new peak is observed. This proves that the chromium oxide was doped into the zinc oxide lattice without remarkable change in its structure. Very few changes were observed in the cell parameters of zinc oxide as a result of being doped with chromium oxide. As Table 1 indicates, these changes exist in *α* axis and *c* axis compared with pure zinc oxide (ZnO-X, X = 2, X = 5 mole% chromium oxide).

**Table 1:** Cell parameters of Cr:ZnO NPs.

Catalyst	α (Ȧ)	ϲ (Ȧ)	α:ϲ ratio	V (Ȧ^3^)	Reference
Pure ZnO	3.249	5.207	0.6239	47.60	51
Used ZnO	3.2525	5.2166	0.6241	47.88	Present work
2 mole% Cr:ZnO NPs	3.253	5.212	0.6241	47.78	Present work
5 mole% Cr:ZnO NPs	3.254	5.213	0.6242	47.79	Present work

Doping often increases the lattice parameters and hence raises cell volume of the nanoparticle. Pure zinc oxide has a cell volume of 47.6 (Ȧ). Doping zinc oxide with 5 mole% of chromium oxide slightly increased the nanoparticle cell volume to 47.6 (Ȧ). This indicates that chromium with an ionic radius of 0.52 (Ȧ) has replaced zinc with an ionic radius of 0.74 (Ȧ)^47^. The results of XRD for the two types of nanoparticle (Cr:ZnO NPs type I and II) demonstrated that crystallite characteristics of these nanoparticles have been retained and chromium oxide has been properly doped in zinc oxide. The maximum diffraction intensity was related to plate 011.

**Figure 2:**
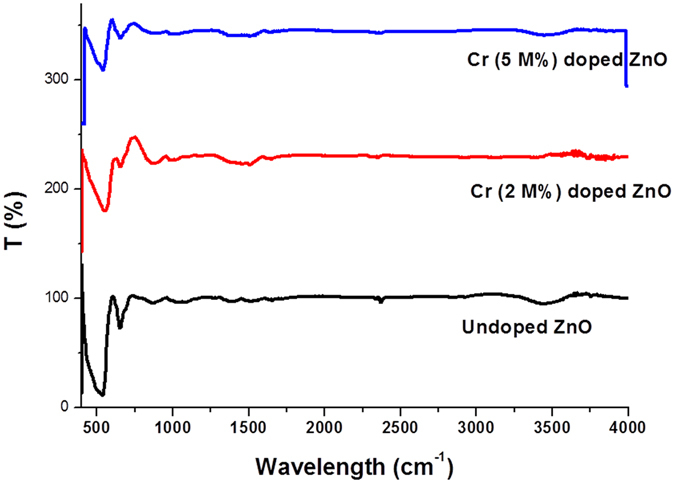
FITR spectrum obtained from zinc oxide nanoparticles doped with chromium oxide type 2.

Functional groups available in the surface of synthesized Cr:ZnO NPs were determined by Fourier transform infrared spectroscopy (FTIR). In Fig. 2, FTIR spectrum shows some peaks due to the presence of the reactive chemically fixed on the surface of nanoparticles. It can be concluded that chromium oxide, which has been added to zinc oxide as dopant has caused some changes during synthesis. It has an organic cover at the surface, which can cause some changes at the surface of nanoparticles. Available peaks at regions around 1516 and 3400 cm^−1^ show the presence of NH_2_ and O-H in the external band of zinc oxide nanoparticles doped with chromium oxide. The presence of a prolonged peak in the area of 536 cm^−1^ is due to doped Cr-O^32^. Moreover, some peaks have been formed in the area around 871 and 651 cm^−1^, which indicate the presence of C-H bonds. As FTIR spectrum demonstrates, there are some peaks in the obtained spectrum showing C-H and =C-H functional groups. They also show that a very small amount of carbon-hydrogen functional group is available in the structure of this nanoparticle because of the effect of *n*-butylamine^48^.

**Figure 3:**
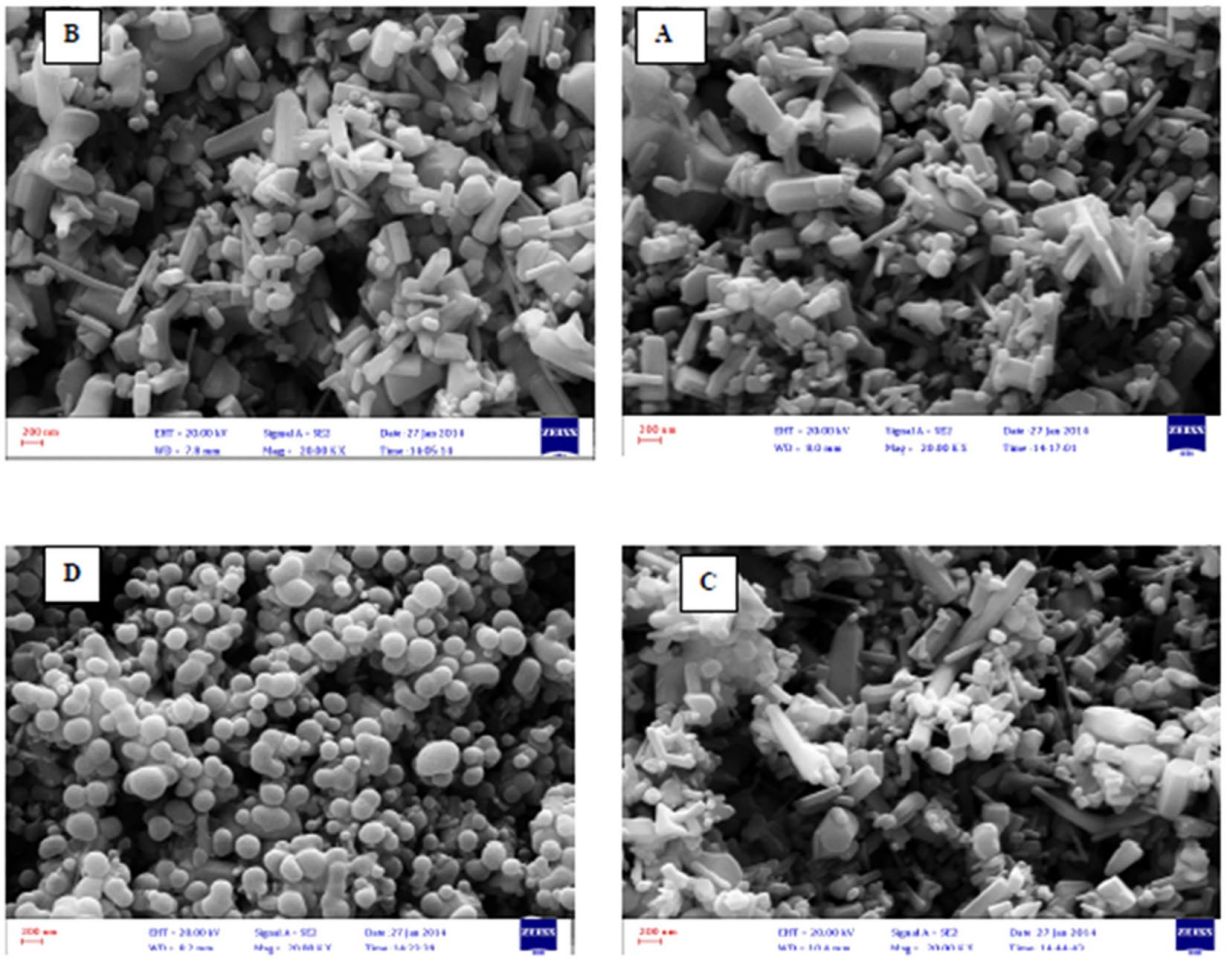
SEM image, (**A**) Powder of zinc oxide nanoparticles doped with chromium oxide; (**B**) A layer of zinc oxide nanoparticles doped with immobilized chromium oxide; (**C**) Three layers of zinc oxide nanoparticles doped with immobilized chromium oxide; (**D**) Zinc oxide nanoparticles doped with immobilized chromium oxide after the photocatalyst process.

**Figure 4:**
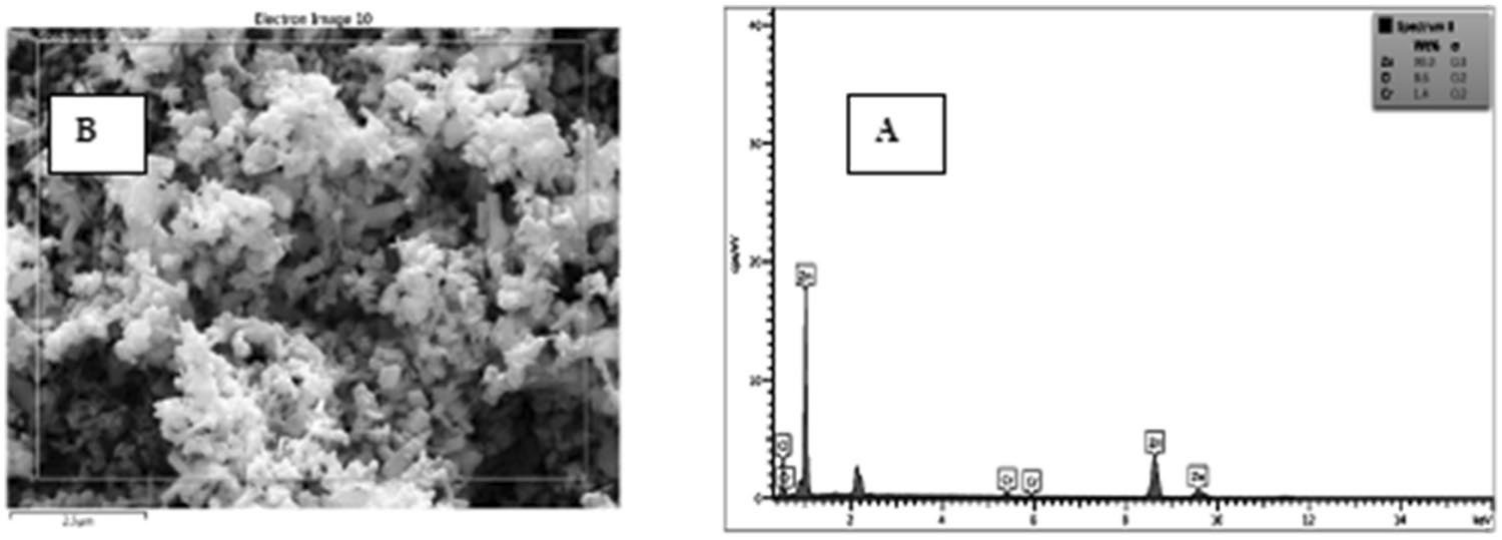
EDX diagram based on the powder of zinc oxide nanoparticles doped with chromium oxide.

**Figure 5:**
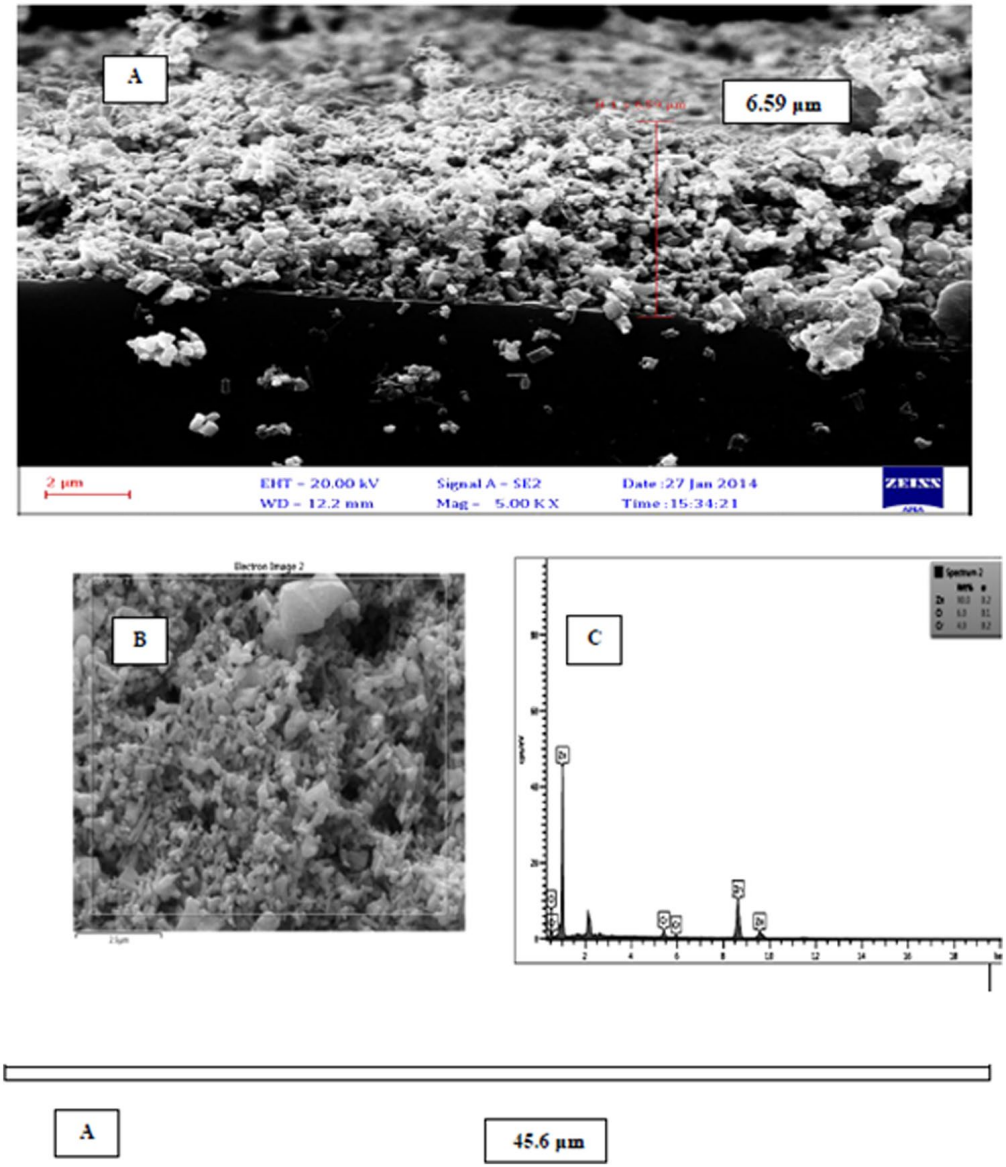
(**A**) Cross sectional SEM image; (**B**) Surface based SEM image; (**C**) EDX diagram based on a layer of doped zinc oxide nanoparticles immobilized on glass plates.

**Figure 6:**
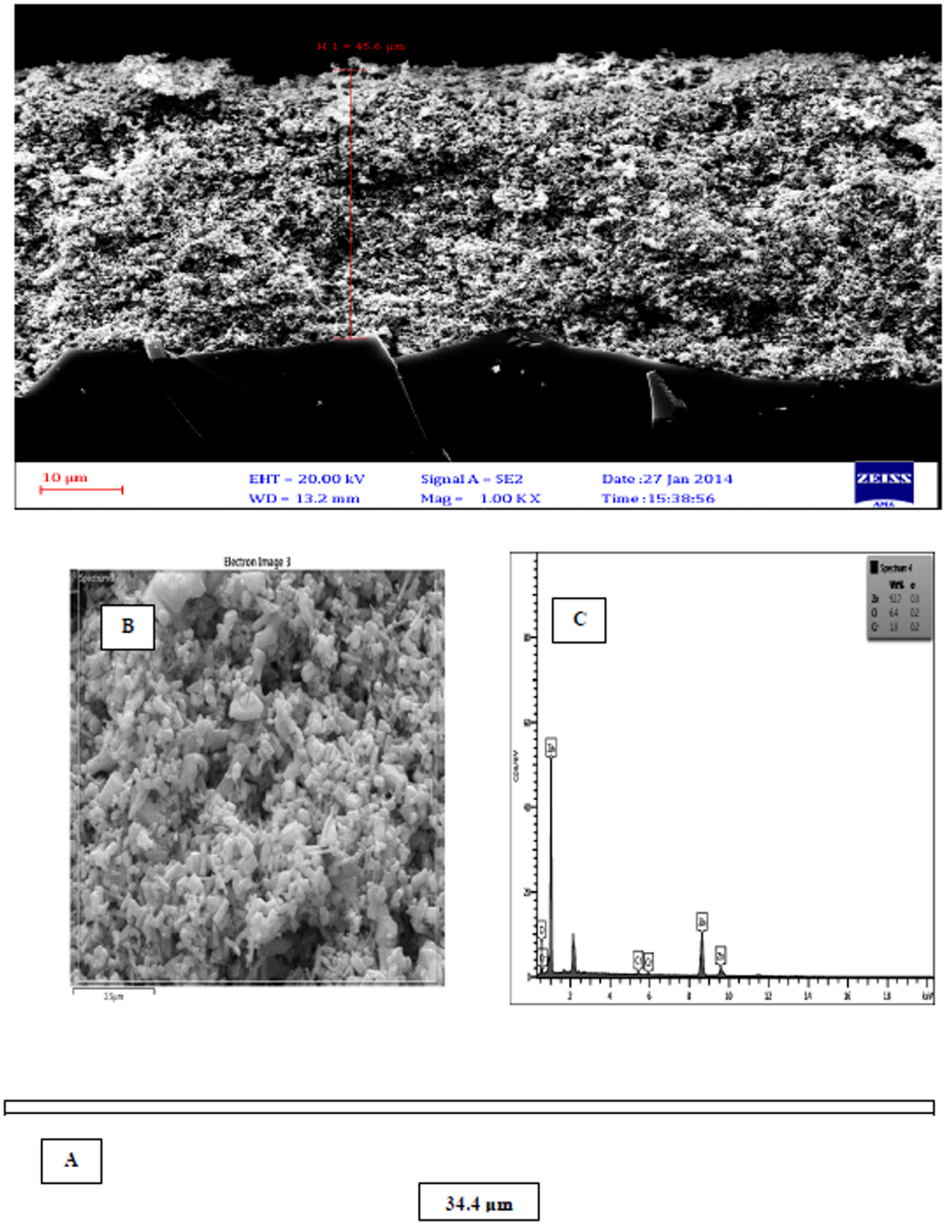
(**A**) Cross sectional SEM image; (**B**) Surface based SEM image; (**C**) EDX diagram based on three layers of doped zinc oxide nanoparticles immobilized on the glass plate.

**Figure 7:**
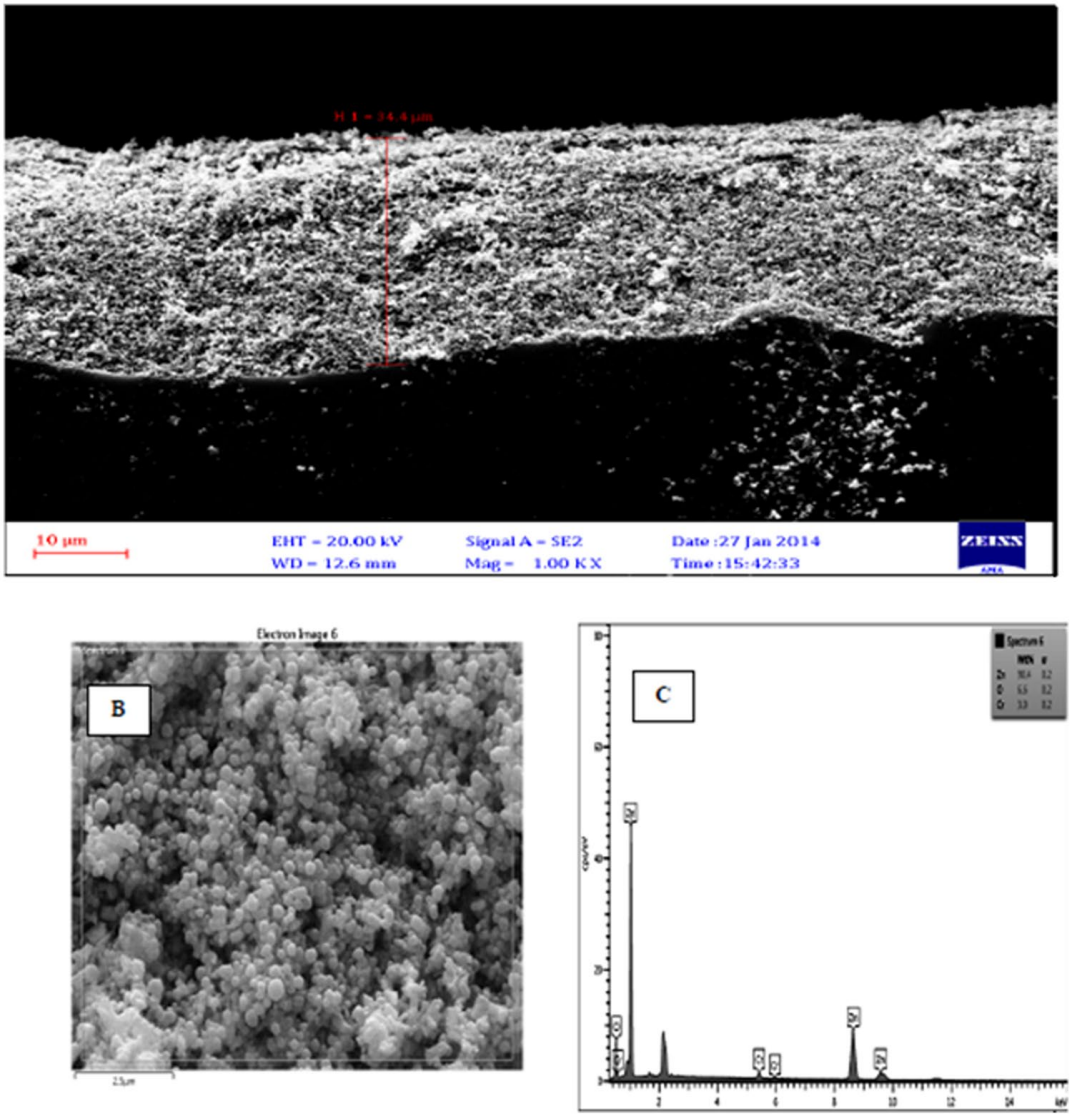
(**A**) Cross sectional SEM image; (**B**) Surface based SEM image; (**C**) EDX diagram based on doped zinc oxide nanoparticles immobilized on the glass plate after the oxidation process.

Figure 3 shows SEM images of zinc oxide nanoparticles doped with chromium oxide in both powder and immobilized forms with one and three layers of synthesized nanoparticles before and after photo-oxidation with a zoom of 2000. At this scale, the size of zinc oxide nanoparticles doped with chromium oxide is 64 nanometers. It was also observed that nanoparticles had a homogenous shape and the average diameter of nanoparticles was 60 to 160 nanometers. The results of this analysis indicate that powder and immobilized zinc oxide nanoparticles have retained their hexagonal structure and possess a proper homogeneous porosity. It was also observed that immobilization has not changed the structure of nanoparticles. As a result, using surfactant for surface modification leads to less agglomeration^33^. The new morphology of zinc oxide nanoparticles made them suitable for photodegradation. Moreover, the synthesized nanoparticles have acceptable degradation and hydrophilic properties, which make them suitable for photodegradation of organic pollutants found in industrial wastewater^49^. Figure 4 illustrates the EDS diagram of the powder form of synthesized nanoparticles. Accordingly, zinc (Zn), chromium (Cr), and oxygen (O) are present in the structure of these nanoparticles. SEM images from the cross-section of the sand-blasted glass plates on which nanoparticle immobilization had been performed reveal the diameter and thickness of different layers of synthesized nanoparticles. As illustrated in Fig. 5, SEM images showed that the thickness of a layer of immobilized nanoparticles was around 6.6 μm. Furthermore, the EDS diagram prepared based on the layer of immobilized zinc oxide shows that the immobilized nanoparticles are pure. On the other hand, Fig. 6, which is based on the SEM image, shows that the thickness of three layers of immobilized nanoparticles is 45.5 μm. EDS diagrams prepared based on the immobilized zinc oxide layer show the presence of zinc (Zn), chromium (Cr), and oxygen (O). Finally, after the photocatalytic reaction for aniline removal, SEM image was obtained from the surface and section of glass plates in order to study the structure and thickness of the immobilized Cr:ZnO nanoparticles. As the SEM image illustrates in Fig. 7, after the photo-oxidation process, the thickness of immobilized nanoparticles became 34.4 μm. This insignificant reduction of thickness could be attributed to the washing and removing less stable nanoparticles. In addition, the EDS analysis performed after photo-oxidation shows that the structure and available elements of nanoparticles did not change and still there is zinc (Zn), chromium (Cr), and oxygen (O) in their structure. However, XPS analysis could give a better understanding and more accurate data on whether chromium is doped in the form of metal or not. The authors did not have access to such instrument to carry out additional supporting analysis.

**Figure 8:**
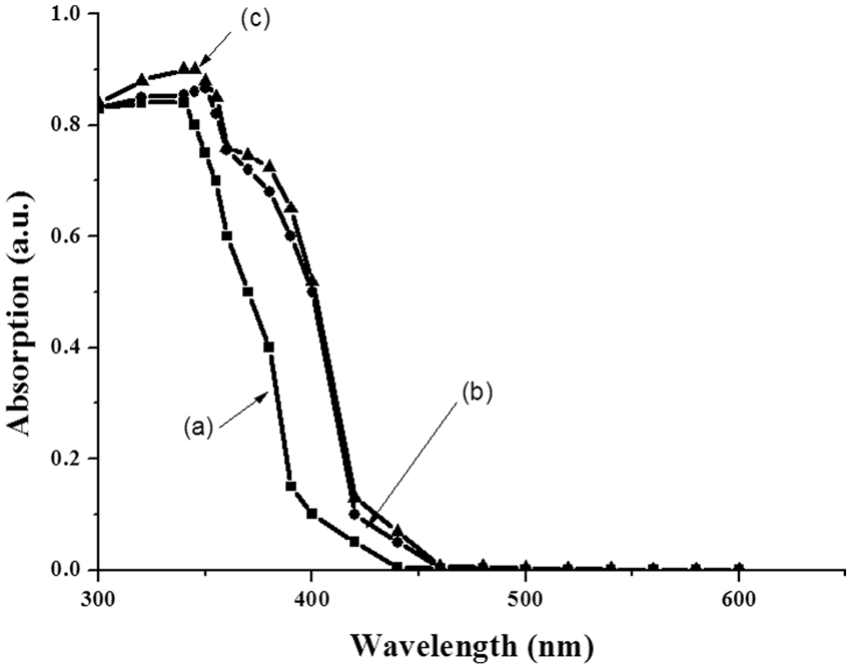
The effect of doping on energy band of zinc oxide nanoparticles doped with chromium oxide. (**A**) Pure powder of zinc oxide; (**B**) Zinc oxide doped with chromium oxide type 1; (**C**) Zinc oxide doped with chromium oxide type 2.

Figure 8 illustrates UV absorption spectrum analysis. As can be seen, the effect of doping with UV absorption spectrum was studied over the wavelength of 200–600 nm. In the area of 350–400 nm, there was an absorption peak. According to the results, pure zinc oxide did not show any absorption in the visible light region (400 nm < λ). However, the Cr:ZnO nanoparticles showed a considerable absorption in the region of longer waves. This is due to the fact that, through doping, the energy gap is reduced and longer waves are absorbed. As a result, because of the absorption of a large proportion of UV-sunlight by the Cr:ZnO catalyst, it is possible to use sunlight and UV-A radiations in photocatalytic processes^50^.

The size of synthesized nanoparticles was determined through Scherrer’s equation^32^. The highest intensity of peaks’ diffraction was on plates 010, 002, and 011.

1 $$D=\frac{k\lambda }{\beta \,\csc \,\theta }$$

In this equation, *k* indicates shape which was considered as 0.89, λ refers to the wavelength in radiation of CuKα_1_ (0.1542 nm), β is the diffraction intensity in various peaks (degree), and θ shows the Bragg angle (half of size of the angles of peaks’ position). The average size of Cr:ZnO nanoparticles was calculated based on Equation 1 (Table 2).

**Table 2:** The size of nanoparticles obtained by Scherrer equation.

D/nm	°θ/	β/°	Surface modifier	Type of nanoparticle
7.1	34.58	0.235	n-butyl amine	5 mol% Cr_2_O_3_:ZnO

**Figure 9:**
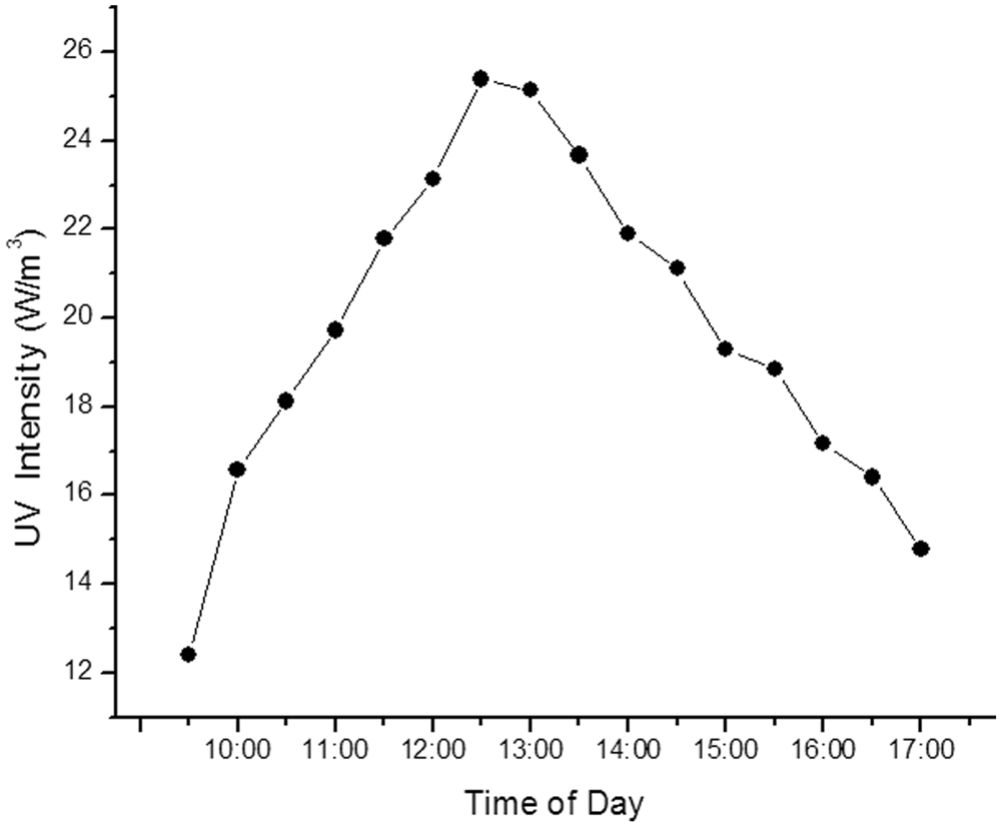
The fluctuation curve of sunlight UV radiation average in different hours of the day.

Figure 9 illustrates the results of the analysis of fluctuations in solar UV (over a week). These fluctuations constitute a normal curve and the maximum UV intensity over the surface (20 w/m^2^) can be found around the noon. Comparatively, the amount of radiated UV during the beginning (before 10 a.m.) and final hours (after 4 p.m.) of the day is reduced by almost half of that at noon. In experiments related to using natural sunlight as the UV source, the system launching time in each stage was 10:30 and lasted until 16:30; hence, the retention time was 6 hours. In this time span, the average radiation intensity of sunlight UV was 20.89 ± 0.57 w/m^2^ roughly equal to the lowest UV intensity of the UV lamp in other stages of the experiment.

**Figure 10:**
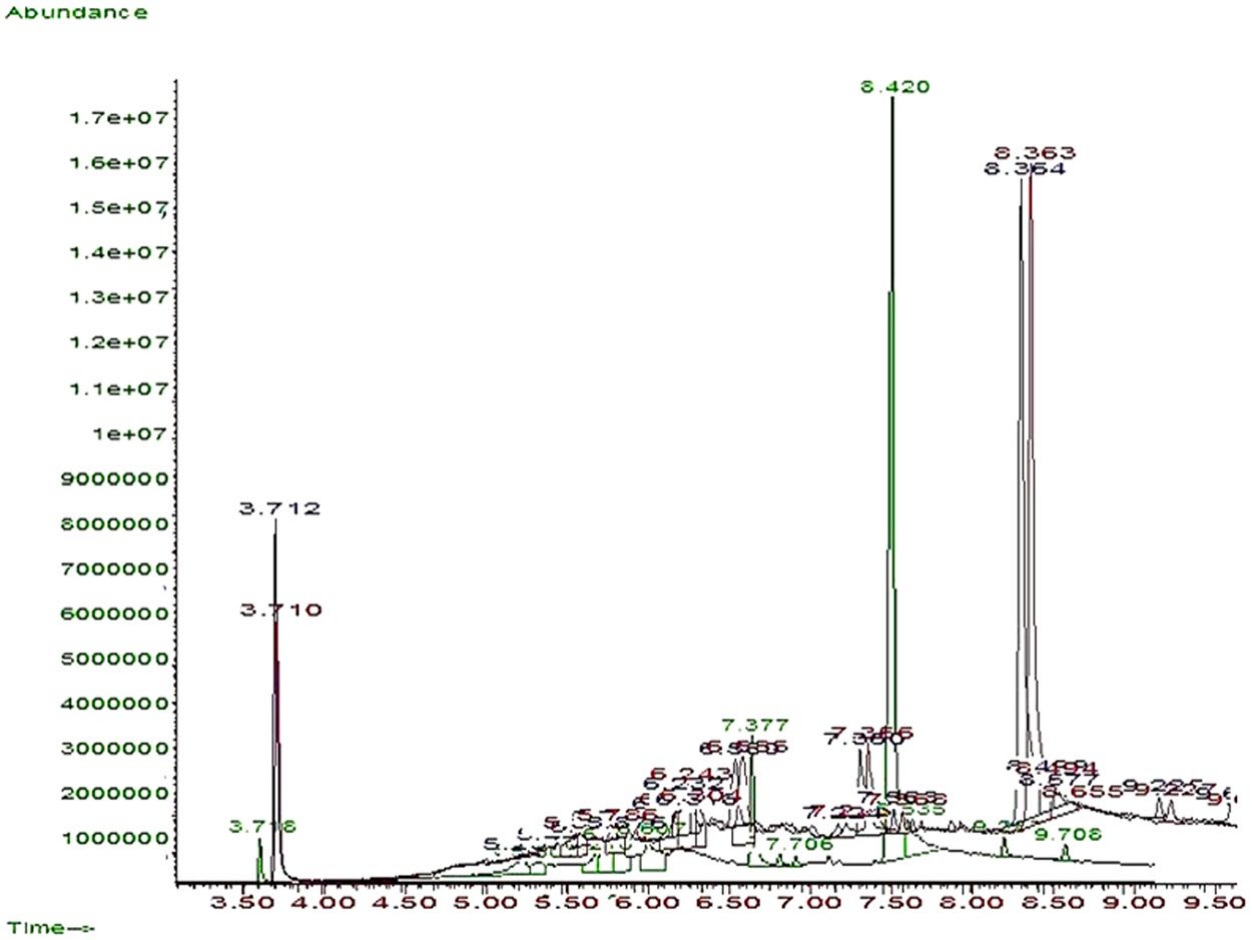
GC-MS Peaks (Aniline concentrations: 150, 200 and 250 mg/L, radiation source: sunlight, pH of the environment: 9, and retention time: 6 hours).

The residual concentration of aniline after photo-oxidation in each stage of the experiment was calculated by GC-MS machine in the light of the area under the peak and by the use of prepared standard solutions^43^. The removal efficiency was also calculated (Table 3). The peaks obtained through aniline analysis by GC-MS machine and their comparison with the peak obtained through standard concentration of aniline revealed that aniline concentration decreased and the compounds were less poisonous. Obtained spectra indicate this issue. Figure 10 shows the spectra obtained from GC-MS machine under applied conditions (aniline concentrations ~150, 200, and 250 mg/L, irradiation source: sunlight, pH of the environment ~9, and retention time ~6 h). Aniline concentration reduced by 93, 82, and 74 percent, respectively. Accordingly, degradation and intermediate byproducts was observed including phenolic compounds, benzoquinone, dodecane and other compounds.

**Table 3:** The primary concentration and the remained Aniline after the photo-oxidation process.

Experiments	Primary Aniline concentration (mg/L)	Radiation source	The average of the concentration of the remained Aniline (mg/L)	The average percent of removed Aniline (%)
1	150	Sunlight	9.91	93.3
2	200	Sunlight	34.78	82.6
3	250	Sunlight	63.75	74.5

**Figure 11:**
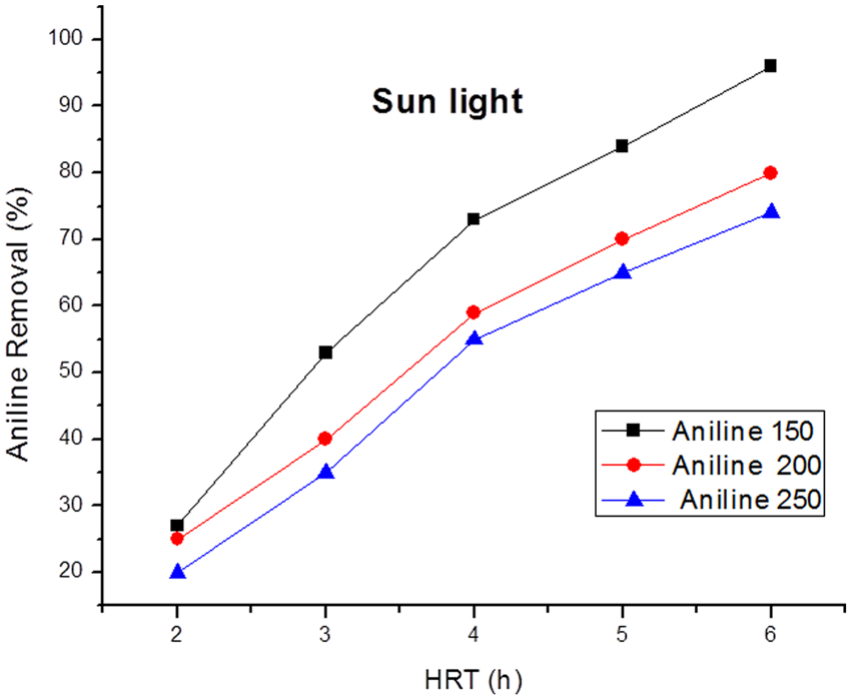
Various concentrations of Aniline under sunlight radiation.

Figure 11 shows the influence of sunlight radiation and visible light on the removal of aniline as a pollutant. As the primary concentration of aniline increases, the amount of removal is reduced. In order to activate the photocatalytic process, the amount of energy should be equal or more than the energy gap of zinc oxide nanoparticles, so that the electrons of valence band can be excited and moved to the conduction band. This energy, which equals 3.2 eV, is the minimum threshold intensity of radiation for activating and starting the photocatalytic process^51^. In order to examine the relationship between radiation intensity and the primary aniline concentration, one way ANOVA was used. The results demonstrated the presence of a significant relationship (P < 0.05).

The results of the experiments related to the effect of pH on the efficiency of aniline removal revealed that zinc oxide is solved in an acid environment with a pH over 13. In the main experiments for photocatalytic removal of aniline in this research, pH of the aqueous environment was controlled to avoid solving Cr:ZnO nanoparticles. The best pH for removing aniline was found to be 9. Ayati *et al*. reached similar findings with regard to pH^52^. Retention time is one of the influential variables in increasing performance efficiency of photocatalytic systems. In this research, for removing aniline through photocatalytic process of Cr:ZnO nanoparticles, the hydraulic retention times were 2, 4, and 6 hours. It was observed that as the hydraulic retention time increased, the efficiency of aniline removal went up. Thus, the optimal hydraulic retention time was 6 hours. The result of the pretest showed that increasing the amount of retention time would lead to better efficiency of aniline removal. Increasing the retention time did not reduce the process of electron excitement of Cr:ZnO nanoparticles and the production of hydroxyl free radicals; however, due to the formation of intermediate organic compounds as a result of aniline degradation, some of the produced free radicals were used for degrading these compounds and, therefore, more time is required to degrade aniline. Ayati *et al*.’s study focused on phenol oxidation by zinc oxide nanoparticles immobilized on concrete. They found that, in order to have a removal efficiency of 90%, 5 hours was the best retention time for phenol oxidation with the primary concentration of 50 mg/L, the nanoparticle dose of 80 g/m^2^, and UV lamp power of 32 Watts^53^.

In photocatalytic oxidation of aniline, this pollutant is degraded as a result of oxidation and is converted to harmless or less harmful intermediate compounds. At the last stage, it is converted to water and carbon dioxide. Some of these intermediate compounds are oxalic acid and oleic acid, which are harmless fatty acids^54^.

## Conclusion

Zinc oxide nanoparticles doped with chromium oxide were successfully synthesized under hydrothermal conditions in the presence of *n*-butylamine as a surfactant. Because it is less poisonous and more economical and also reduces nanoparticles’ agglomeration, this surfactant is a suitable surface modifier. Doping zinc oxide with a suitable metal oxide like chromium (Cr^+3^) can reduce the energy gap of nanoparticles and increase the power of nanoparticles in using sunlight for the photocatalytic degradation of pollutants. Compared with UV lamps, the use of sunlight and visible light on large scale is more economical and has less strategic problems. Immobilizing nanoparticles on a surface glass, which is inexpensive and convenient, has a number of advantages; first, all the immobilized particles receive an equal amount of radiation; second, it is not necessary to remove and split catalyst particles; and, third, it prevents the waste of some consumed nanoparticles, the increase of treatment costs, and the need for water pretreatment before applying these particles. The results of characterization revealed that the morphology and crystallite structure of synthesized nanoparticles were retained even after immobilization on the glass bed and the photocatalytic reaction of aniline removal. The synthesized nanoparticles immobilized on the glass bed had the optimal efficiency for removing aniline by the use of sunlight in the retention time of 6 hours and pH of 9. The findings showed that these nanoparticles are able to use sunlight to degrade aniline.
